# Complete genome sequencing and comparative genomic analysis of three donkey *Streptococcus equi* subsp. *equi* isolates

**DOI:** 10.3389/fmicb.2023.1285027

**Published:** 2023-11-01

**Authors:** Yuwei Zhang, FenFen Lv, Yan Su, Huan Zhang, Baojiang Zhang

**Affiliations:** Department of Microbiology and Immunology, College of Veterinary Medicine, Xinjiang Agricultural University, Ürümqi, Xinjiang, China

**Keywords:** *Streptococcus equi*, whole genome sequencing, comparative genomics, virulence, antimicrobial resistance, donkey

## Abstract

**Introduction:**

*Streptococcus equi* subspecies *equi* (*S. equi*) is the causative agent of strangles, which is one of the most common and highly contagious respiratory infectious illnesses in horses. *Streptococcus equi* (*S. equi*) is a horse-specific pathogen that originated from the closely related zoonotic pathogen *Streptococcus equi* subspecies *zooepidemicus* (*S. zooepidemicus*). Despite decades of research, the movement of genetic material across host-restricted diseases remains a mystery.

**Methods:**

Three *S. equi* donkey isolates (HTP133, HTP232, and HT1112) were recently isolated from a strangles epidemic on donkey farms in China’s Xinjiang Province. In this study, we performed a comprehensive comparative analysis of these isolates using whole genome sequencing and compared them to the published genomic sequences of equine strain *S. equi* 4047 to uncover evidence of genetic events that shaped the evolution of these donkey *S. equi* isolates’ genomes.

**Results:**

Whole genome sequencing indicated that both strains were closely related, with comparable gene compositions and a high rate of shared core genomes (1788-2004). Our comparative genomic study indicated that the genome structure is substantially conserved across three donkey strains; however, there are several rearrangements and inversions when compared to the horse isolate *S. equi* 4047. The virulence factors conveyed by genomic islands and prophages, in particular, played a key role in shaping the pathogenic capacity and genetic diversity of these *S. equi* strains. Furthermore, we discovered that the HT133 isolate had a strong colonization ability and increased motility; the HT1112 isolates had a significantly higher ability for antimicrobial resistance and biofilm formation, and the HT232 isolate gained pathogenic specialization by acquiring a bacteriophage encoding hyaluronate lyase.

**Discussion:**

In summary, our findings show that genetic exchange across *S. equi* strains influences the development of the donkey *S. equi* genome, offering important genetic insights for future epidemiological studies of *S. equi* infection.

## Introduction

*Streptococcus equi* subspecies *equi* (*S. equi*) is the causative agent of strangles, which is one of the most commonly diagnosed and feared serious, highly contagious respiratory infectious equids diseases in the world, with morbidity rates of up to 100% (Waller, 2013; Cordoni et al., 2015). Outbreaks impose a significant financial burden on the equine industry and horse owners, as well as raising concerns about horse health and welfare (Boyle et al., 2018).

As a result, initiatives to enhance diagnosis and prevention are critical to the equestrian sector. The use of molecular biological approaches has tremendously benefited epidemiological research, such as tracking the epidemic (Bertelli and Greub, 2013). Furthermore, next-generation sequencing methods like whole genome sequencing (WGS) and comparative genomic analysis have recently become very strong tools for examining the increase and loss of particular virulence and antibiotic resistance genes. Gene gain by horizontal acquisition is a critical element in the establishment of novel pathogenic *streptococci* strains (Harris et al., 2015). Receiving bacteria may gain an instant selective advantage by acquiring antibiotic resistance mechanisms or producing novel virulence activities. Furthermore, such research provides information on the proclivity of certain isolates for invasion.

Previous studies have contributed to our understanding of the genetic and pathogenic characteristics of a couple of *S. equi* strains isolated from horses. Holden et al. (2009) sequenced the complete genome sequences of *S. equi* 4047 and *S. zooepidemicus* H70, analyzed their general genome features, and compared the genomes from around the world to uncover genetic evidence of their evolution events. Researchers (Alber et al., 2005; Paillot et al., 2010) found that the evolution of *S. equi* from an ancestral *S. zooepidemicus* is associated with the acquisition of four prophage-encoded virulence genes: *seeI*, *seeL*, *seeM*, and *seeH*. So far, only a limited number of genomic sequences of equine *S. equi* strains have been sequenced, and genomic resources for donkey isolates are still not available. It is not yet known how the donkey isolate affects the evolution and exchange of genetic material of these host-restricted pathogens.

Whole genome sequencing (WGS) and a subsequent comparative genomic analysis can be used to understand the movement of virulence and other host adaptation genes between *streptococci* isolates from different hosts. Furthermore, WGS results could help the prevention and control of strangles outbreaks. Thus far, there is no publicly available data regarding the sequences of *S. equi* donkey isolates recovered in China, which has limited our comprehensive and systematic understanding of these isolates’ evolution and fitness in donkey hosts.

According to the 16S rRNA gene and physiological and biochemical studies, we have found three *S. equi* strains designated *S. equi* HTP133, HTP232, and HT1112 (ST-179) recovered from donkeys with strangles in Xinjiang, China, in 2020. We report on the WGS, virulence factors, and antibiotic susceptibility of three *S. equi* donkey isolates from Xinjiang, China, in order to investigate genetic diversity and virulence features. Following that, we compared the genomes of these donkey strains isolated in China to the genomes of *S. equi* 4047, a virulent strain isolated from a horse with strangles in the New Forest, England, in 1990 (Kelly et al., 2006), to better understand the evolution of within-host adaptation and shed new light on the evolution of this donkey *S. equi* lineage toward host restriction.

## Materials and methods

### *S. equi* isolates and culture conditions

Isolates of *S. equi* Lancefield group C were represented by Se HT1112 (2019), Se HTP133 (2020), and Se HTP232 (2020). *S. equi* was isolated from donkey samples of different farms during strangles outbreak in the Chinese region of Xinjiang and has been typed as ST-179 by multi-locus sequence typing (MLST). At 37°C, all strains were cultivated overnight in Todd-Hewitt Broth (THB) containing 5% sheep blood (OXOID, Basingstoke, Hampshire, England).

### Genomic DNA extraction

The genomic DNA from *S. equi* isolates was extracted from bacterial cell pellets using a TIANamp Bacteria DNA Kit (Tiangen, Beijing, China) according to the manufacturer’s instructions. DNA was resuspended in nuclease-free water and quantified with a NanoVue spectrophotometer (GE Healthcare, Little Chalfont, United Kingdom). DNA quality and RNA contamination were assessed by electrophoresis with 0.8% agarose gels.

### Whole genome sequencing, functional genome annotation, and comparative genomic analysis

The whole genome was sequenced at Shanghai Majorbio Bio-pharm Technology Co., Ltd., (Shanghai, China) using a single-molecule real-time (SMRT) sequencing platform, PacBio RS II (version 2.3.0, Pacific Biosciences, USA), and Illumina sequencing platforms.

The clean data were assembled using SOAP denovo2 for draft genomes and Canu or SPAdes v.3.8.0 for complete genomes. The quality-controlled reads were *de novo* assembled using the RS Hierarchical Genome Assembly Process protocol by SMRT Analysis. Genome annotation coding sequence (CDS) predictions and annotations were performed using RAST (Overbeek et al., 2014), Glimmer v3.02 (Delcher et al., 1999), and the predicted gene sequences were translated and aligned against the National Center for Biotechnology Information Non-Redundant Protein Database (NR) database, the Gene Ontology database, the Clusters of Orthologous Groups (COG) (von Mering et al., 2003), Swiss-Prot, Pfam, and the Kyoto Encyclopedia of Genes and Genomes database (Kanehisa et al., 2012; Kanehisa and Goto, 2000). The circular genome map was constructed with CGView Server (Grant and Stothard, 2008). Genomic islands (GIs) were predicted by IslandViewer 4 (Bertelli et al., 2017).

PHASTER (Arndt et al., 2016) and IslandViewer 4 (Bertelli et al., 2017) were used to identify prophages and gene islands, respectively. The CRISPR Recognition Tool v1.1 (Bland et al., 2007) was used to predict CRISPR. The Circos program was used to generate circular genomes based on anticipated ORFs, rRNA, tRNA, prophages, gene islands, and GC skew information (Krzywinski et al., 2009). Stothard and Wishart (2005) used CGView to create the genome atlas. Resistance Gene Identifier software was used to annotate ARDs (Antibiotic Resistance Datas) against the Comprehensive Antibiotic Resistance Database (CARD) (Alcock et al., 2020).

The reference sequences of these three *S. equi* strains (HT1112, HTP133, and HTP232) were the entire genome sequences of strain *S. equi* 4047, and the BLAST Ring Image Generator (BRIG) (Alikhan et al., 2011) was used for genome-wide comparison to build a circular genomic map. Darling et al. (2010) used progressive Mauve to align the four *S. equi* strains’ genomes. The Majorbio Cloud Platform 3′s online tool OrthoMCL 2 was utilized for comparative analysis to detect the common and unique genes contained by various isolates.

### Pan-genome and core genome analysis

The pan-genome and core genome analyses were conducted as described by Medini et al. (2005). For pan-genome analyses, starting with a single genome, genomes were added in a randomized order without replacement at each fixed number of genomes. The sizes and numbers of the pan-genome and core genomes were calculated as a function of the number of genomes sequentially included.

### Antimicrobial susceptibility testing

Antimicrobial susceptibility testing was performed by the disk diffusion method according to Clinical and Laboratory Standards Institute [CLSI] (2020) guidelines with Se HT1112, HTP133, and Se HTP232. Twenty-one antibiotics, including amoxicilin, ampicillin, cefuroxime, ceftiofur, cefoxitin, penicillin, gentamicin, streptomycin, erythromycin, clarithromycin, doxycycline, oxytetracycline, levofloxacin, tetracycline, norfloxacin, enrofloxacin, ciprofloxacin, sulfafurazole, sulfadiazine sodium, rifampin, and clindamycin, were selected to determine antimicrobial susceptibility. The *Escherichia coli* strain ATCC 25922 was used as the control strain for susceptibility studies.

### Biofilm formation assay

The crystal violet technique was used for the biofilm experiment, as previously described (Wang et al., 2011; Zarankiewicz et al., 2012). An *S. equi* (colony HT1112, HTP133, and HTP232 strains) was injected into 5 mL of THB and cultured overnight at 37°C with shaking. Cultures were diluted 1:100 with new THB broth containing 1% fibrinogen and incubated at 37°C without shaking for 24 h. The positive control was *S. equi* spp. (Se HT1112, HTP133, and Se HTP232 strains) growing on a THB medium containing 1% fibrinogen. The negative control was uninoculated culture media containing 1% fibrinogen. The supernatant with unadhered bacteria was aspirated, and the wells were washed three times with 200 μL of sterile PBS. The wells were stained for 10 min with 200 μL 0.1% (w/v) crystal violet, then rinsed five times with 200 μL PBS to eliminate unbound crystal violet dye. Following the drying of the plate, biofilm-adsorbed crystal violet was resolubilized by adding 150 μL 95% (v/v) ethanol to each well and incubating for 15 min. The absorbance of each well was measured at 595 nm to evaluate biofilm levels. All tests were carried out in triplicate.

### Virulence experiments and growth kinetics

Animal experiments were approved and conducted in strict compliance with the guidelines and regulations of the University of Xinjiang Agricultural University Animal Care and Use Committee.

Before inoculation of mice, cultures of Se HT1112, HTP133, and Se HTP232 strains were collected at 37°C until the logarithmic growth phase was reached, washed twice in THB, and adjusted to the appropriate dose. Experimental mice were injected subcutaneously with 5 × 10^8^, 1 × 10^9^, 2.5 × 10^9^ CFU per mouse. Control mice were injected with sterile PBS. The mice were observed for 14 days for survival status, and the survival differences were plotted by Kaplan–Meier curves and evaluated using the log-rank test. The liver and spleen of experiment mice were homogenized and diluted properly for bacterial load investigation, and *S. equi* colonies were counted on TH medium.

A single colony of *S. equi* (Se HT1112, HTP133, and Se HTP232 strains) was injected into 5 mL of THB and cultured for 24 h with shaking at 37°C. To assess growth, the OD 600 was measured and recorded every 2 h. The sequences utilized for comparative genomic analysis were from *S. equi* subsp. *zooepidemicus* strain 4047 (FM204883) (Holden et al., 2009).

### Evaluation of hyaluronidase activity

Cultures of Se HT1112, HTP133, and Se HTP232 were cultivated in the THB medium to mid-logarithmic phase. Five milliliters of OD600 = 0.4 culture were centrifuged, the supernatants discarded, and the bacteria pellets resuspended in 100 μl of deionized water. The cells were then treated with 0.5% hyaluronic acid (HA) for 30 min at 40°C before being boiled for 3 min to inactivate them. Hyaluronidase activity was determined by adding 400 μL of DNS solution (Sigma), boiling for 5 min and measuring the optical density at 540 nm using a BioTek ELx808 Absorbance Microplate Reader (BioTek, Winooski, United States).

### Nucleotide sequence accession number

The complete genome sequences of three donkey *S. equi* strain (HT1112, HTP133 and HTP232) have been submitted to the GenBank database with accession number CP133957, CP133955, CP133956.

### Statistical analysis

Each experiment in this study has been performed for at least three biological replicates. Data were analyzed with the software SPSS version 16.0 and GraphPad Prism version 7 (GraphPad, La Jolla, CA, United States). Data are presented as mean ± SE or as geometric mean. For each assay, we applied a one-way ANOVA with Tukey’s multiple comparisons test to assess the differences between groups. The survival data between mouse groups were analyzed with the log-rank test (Mantel–Cox). A value of *p* < 0.05 was considered statistically significant.

## Results

### Genome assembly and features of *S. equi* HT1112, HTP133, and HTP232

**FIGURE 1:**
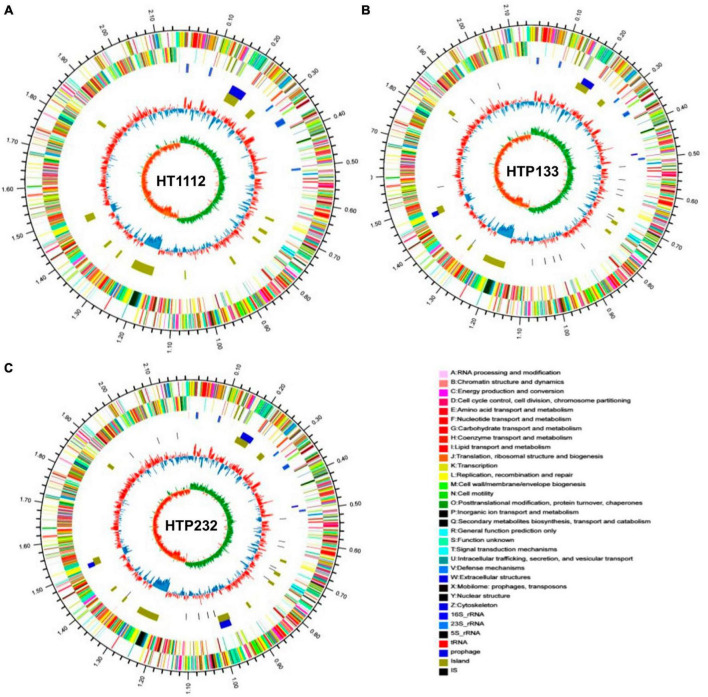
Schematic circular diagrams of three donkey *S. equi* isolates **(A)** HT1112, **(B)** HTP133, and **(C)** HTP232. The outer circles showed the scale marks of the genome. Circles 1 and 2 displayed the protein-coding genes on the forward strand and reverse strand, respectively. Circle 3 displayed the tRNA and rRNA genes on the forward strand. Circle 4 displayed the tRNA and rRNA genes. Circles 5, 6, and 7 displayed the prophage, genome island, and insert sequence, respectively. 16S rRNA (dark blue), 23S rRNA (pale blue), 5S rRNA (green), tRNA (red), prophage (purple), and island (yellow). The prophecies are displayed in blue. The brown color represents the genomic island. The genome map was made using Circos v0.64 (http://circos.ca/).

Features of the three newly sequenced and four published genomes of *S. equi* are shown in Table 1. The sizes of three *S. equi* genomes range from 2.15 Mb to 2.19 Mb, and the GC contents range from 41.30 to 41.36%. The predicted numbers of open reading frames (ORFs) range from 2,010 (1,824,747 bp, HTP133) to 2,223 (1,872,897 bp, HTP232), with average lengths ranging from 842 bp to 907 bp. The coding genes function (COG) analysis showed that the gene numbers of cellular component (823), molecular function (1,372) and biological process (1,160) in Se HT1112 were more than those of Se HTP133 (7,351,262,803) and HTP232 (7,441,264,806). The genomes of three isolates were examined for genome architecture, chromosomal sequences, and mobile elements such as GIs, prophage regions, and insertion elements (Figure 1). CRISPR is a unique family of direct repeat DNA sequences that widely exist in prokaryotic genomes. There are seven predicted CRISPR arrays in the Se HT1112 and HTP133 genomes and eight CRISPR arrays in the Se HTP232 strain. Features of the three newly sequenced genomes of *S. equi* are shown in Table 1.

**TABLE 1:** Basic genomic characteristics of the strains.

Features	HT1112	HTP133	HTP232
Chromosome (bp)	2,156,526	2,150,351	2,191,031
Coding genes	2,167	2,010	2,223
Gene size (bp)	1,837,221	1,824,747	1,872,897
Gene average length (bp)	847.82	907.83	842.51
G + C content (%)	41.97	41.97	41.92
tRNA	77	67	67
rRNA	21	18	18
16s rRNA	7	6	6
23s rRNA	7	6	6
5s rRNA	7	6	6

**FIGURE 2:**
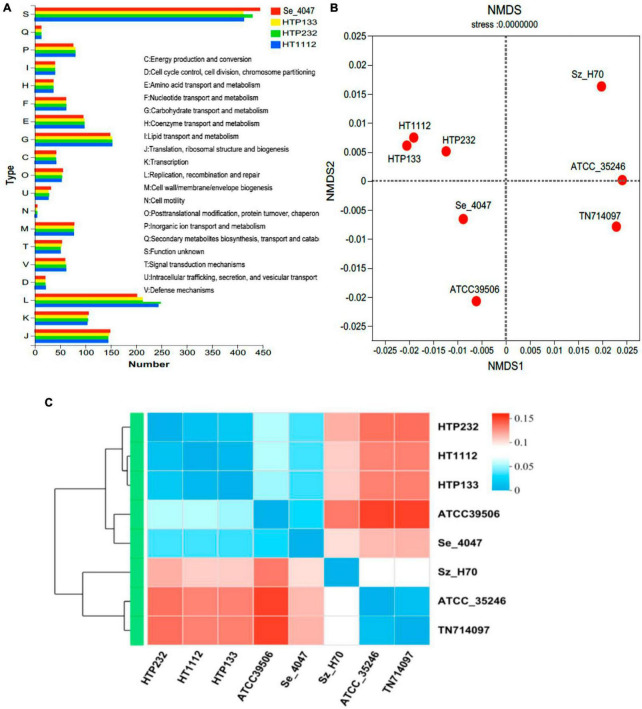
Analysis of the COG function of *S. equi* strains HT1112, HTP133, and HTP232 and comparison with the other 5 reference strains. **(A)** COG function comparison of HT1112, HTP133, and HTP232 with Se 4047 (GCF000026). **(B)** Non-metric multidimensional scaling (NMDS) analysis of three isolates (HT1112, HTP133, and HTP232 genomes) and five reference strain genomes based on COG function. The NMDS results revealed genome clustering in three isolates relative to these groups that were used as reference strains. **(C)** Heatmap based on the COG function showing evolution relations with respect to the COG function of six *streptococci* reference strain genomes and three isolates (HT1112, HTP133, and HTP232 genomes).

Identification of prophages is important for the study of the genomes of the *S. equi* strains and their genetic potential. Prophage element analysis revealed that the genome of HTP232 harbors four prophages with sizes ranging from 8.6 to 17.5 kb, while the other two strains, HTP133 harbors three prophages and HT1112 harbors one prophage (Table 2). Seven *S. equi* and *S. zooepidemicus* isolates with different COG function classifications were selected for heatmap evaluation. Non-metric multidimensional scaling (NMDS) (Figure 2B) and heatmap (Figures 2A, C) based on COG function suggesting a high degree of relationship was observed for isolates HTP133, HT1112, HTP232, and these three donkey isolates have a close relationship with other two *S. equi* isolates 4047 and ATCC39506 (Figure 2C).

**TABLE 2:** Annotated table of gene function results.

Strains	GO	COG	KEGG	NR	Swiss-port	Prophage	Crispr-Cas	GI
HT1112	1,687	1,706	1,197	2,166	1,506	1	7	12
HTP133	1,527	1,708	1,202	2,010	1,509	3	7	10
HTP232	1,540	1,731	1,200	2,151	1,516	4	8	12

### Detection of putative virulence genes and comparison of growth

**FIGURE 3:**
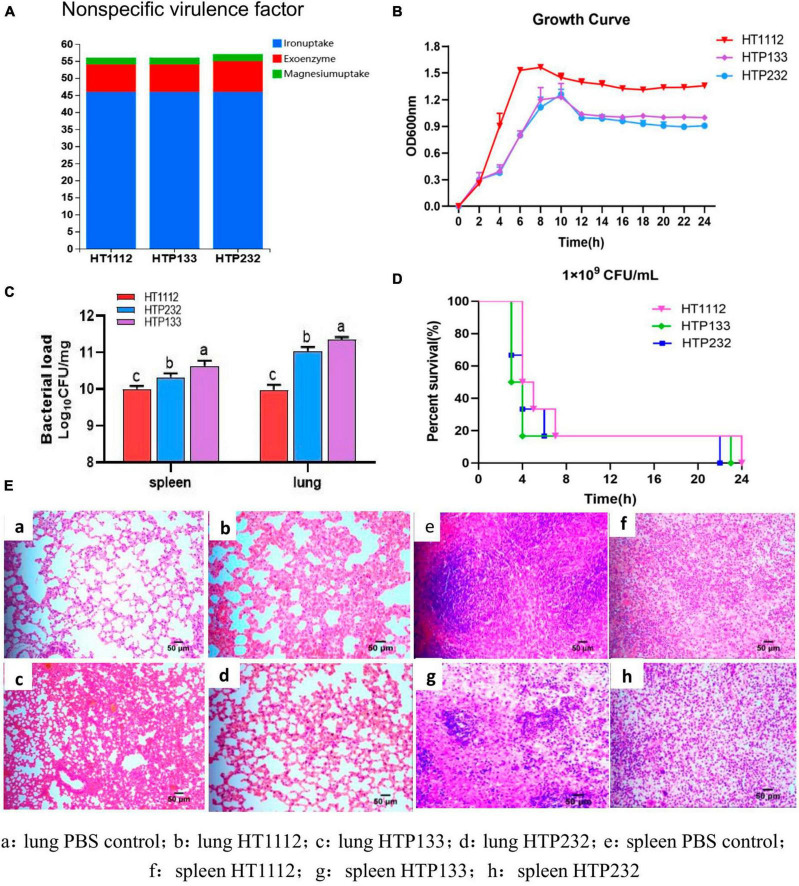
The virulence analysis of three isolates (HT1112, HTP133, and HTP232). **(A)** Distribution of non-specific virulence genes in three *S. equi* isolate genomes. **(B)** Growth curves of these three *S. equi* isolates. HT1112, HTP133, and HTP232 were cultured in THB broth at 37°C. The OD600 nm of the culture was measured every 2 h. The data represent the means and standard deviations of the results of three independent experiments. **(C)** Survival rates of *S. equi* strains, HT1112, HTP133, and HTP232, in BALB/C male mouse models. The groups of 5–6-week-old mice were subcutaneously inoculated with 1.0 × 10^9^ CFU of *S. equi* isolates HT1112, HT232, and HT133 and monitored for 24 h. **(D)** At 24 h post-infection, the mice were sacrificed, and their spleens and lungs were harvested and homogenized to measure the CFUs of bacterial colonization. Colonies were expressed as Log10CFU/mg, and the results are shown as the mean of three mice in each group (SD, *p* < 0.001; *p* < 0.01; *p* < 0.05. CFU = colony-forming unit). The differences between survival curves were evaluated using the log-rank test (*n* = 6 mice). **(E)** Lung and spleen tissues of mice subjected to HE staining. There is a significant difference (*p* < 0.05) between numbers with different letters; no significant difference (*p* > 0.05) exists between numbers with the same letter.

We found 169 (HT1112), 169 (HTP133), and 170 (HTP232) potential virulence factors (VFs) that were involved in 13 functions, including adherence, anti-phagocytosis, exoenzyme, invasion, iron uptake, regulation, complement protease, magnesium uptake, toxin, antiphagocytosis, serum resistance, stress protein, and secretion system. All three bacteria possess eight non-specific VFs that encode exoenzyme genes. When the VFs of the three strains were compared, we discovered that the exoenzyme-coding gene was only detected in the HTP232 strain and was not present in the other two isolates, HTP133 and HT1112 (Figure 3A).

In growth experiments, the growth rate dynamics revealed different growth rates among the three isolates. The *S. equi* HT1112 isolate had a shorter lag phase and a higher growth rate compared with the other two strains, HTP133 and HTP232 (Figure 3B).

### Virulence-associated phenotypes

The connection between VFs and virulence-associated phenotypes of isolates HT1112, HTP133, and HTP232 was investigated to assess the virulence features. In a mouse infection paradigm, mice given the strains HT1112, HTP133, and HTP232 (1 × 10^9^ CFU) all died within 24 h; the isolate HTP133 was the most deadly of the three. More germs were found in the spleen and lungs of mice infected with HTP133 than with HT1112 or HTP232, suggesting that this strain is particularly virulent and causes the most death and damage in animals (Figures 3C, D). Mice with HT1112, HTP133, or HTP232 infections had their lung and spleen tissues fixed for histopathological investigation (Figure 3E). The HE test showed that the lung tissues had been damaged by the challenge, showing enlarged alveolar septa, and the spleen had shown inflammation and bleeding.

### Resistance genes and phenotypes

**FIGURE 4:**
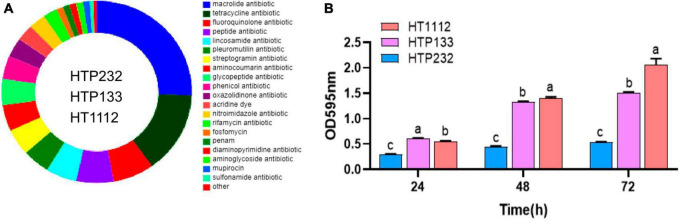
Analysis of the antibiotic resistance phenotype, resistance genes, and biofilm formation of three isolates (HT1112, HTP133, and HTP232). **(A)** Number and distribution of antibiotic resistance genes. **(B)** Biofilm formation by three isolates (HT1112, HTP133, and HTP232) determined by the microliter plate assay after crystal violet staining of bacterial cultures. The strain HT1112 showed higher biofilm formation than HTP133 and HTP232 (*P* < 0.01). The data represent the means and SDs of three independent experiments. There is a significant difference (*p* < 0.05) between numbers with different letters; no significant difference (*p* > 0.05) exists between numbers with the same letter.

We used the CARD to investigate the genomes of the three isolates for antibiotic resistance genes (ARGs), and 172 resistance genes (of 22 types) were found there. These genes were potentially involved in resistance to macrolide, tetracycline, fluoroquinolone, peptide, lincosamide, pleuromutilin, streptogramin, aminoglycoside, glycopeptide, phenicol, oxazolidinone, acridine dye, nitroimidazole, rifamycin, fosfomycin, penam, diaminopyrimidine, aminoglycoside, mupirocin, sulfonamide, and glycylcycline. Among them, 25 tetracycline resistance genes and 44 macrolide resistance genes were discovered in the genomes of the samples (Figure 4A).

All of the isolates were subjected to antimicrobial susceptibility tests, and the results showed that a significant difference in resistance to antimicrobials was observed for these three strains. The HT1112 is resistant to six antimicrobials, including penicillin, cefoxitin, clarithromycin, ciprofloxacin, sulfadiazine sodium, and clindamycin, compared with two antimicrobials (cefuroxime and penicillin) for HTP133 and HTP232 isolates (Table 3).

**TABLE 3:** Antimicrobial resistance phenotype and antimicrobial resistance genes (ARG) of three strains.

Drugs	Phenotype (HT1112)	ARG	Phenotype (HTP133)	ARG	Phenotype (HT232)	ARG
Amoxicilin	S	PBP1a/PBP2x/soxS	S	/	S	/
Ampicillin	S	PBP1a/PBP2x/soxS	S	/	S	/
Cefuroxime	R	PBP1a/PBP2x/soxS	R	/	S	/
Ceftiofur	S	PBP1a/PBP2x/soxS	S	/	S	/
Cefoxitin	S	PBP1a/PBP2x/soxS	S	/	R	/
Penicillin	R	PBP1a/PBP2x/soxS	R	/	R	/
Gentamicin	S	gidB /baes/rpsL	S	baeS/parY	I	baeS/parY
Streptomycin	S	gidB /baes/rpsL	S	baeS/parY	I	baeS/parY
Erythromycin	S	lmrP/efrA	S	efrA/macB/oleC/oleC/mtrA/optrA/lmrC/erm(45)/lmrP/vgaE/optrA	S	efrA/macB/oleC/oleC/mtrA/optrA/lmrC/erm(45)/lmrP/vgaE/optrA
Doxycycline	S	lmrP/efrA	S	efrA/macB/oleC/oleC/mtrA/optrA/lmrC/erm(45)/lmrP/vgaE/optrA	I	efrA/macB/oleC/oleC/mtrA/optrA/lmrC/erm(45)/lmrP/vgaE/optrA
Oxytetracycline	S	rpsJ/adeR/soxS/tetT/lmrP/tetB(P)/soxS/efrA	S	tetA(46)/tetB(46)/tetA(58)/otr(A)/optrA/tetB(P)/tetW/lmrC/tetT/lmrP/vgaE/optrA/adeR	S	tetA(46)/tetB(46)/tetA(58)/otr(A)/optrA/tetB(P)/tetW/lmrC/tetT/lmrP/vgaE/optrA/adeR
Tetracycline	S	rpsJ/adeR/soxS/tetT/lmrP/tetB(P)/soxS/efrA	S	tetA(46)/tetB(46)/tetA(58)/otr(A)/optrA/tetB(P)/tetW/lmrC/tetT/lmrP/vgaE/optrA/adeR	S	tetA(46)/tetB(46)/tetA(58)/otr(A)/optrA/tetB(P)/tetW/lmrC/tetT/lmrP/vgaE/optrA/adeR
Levofloxacin	S	efrA/arlR/arlS/gyrA/efrB/patB/soxS/efrA	S	efrA/arlR/arlS/arlS/patA/patB	S	efrA/arlR/arlS/arlS/patA/patB
Norfloxacin	S	efrA/arlR/arlS/gyrA/efrB/patB/soxS/efrA	S	efrA/arlR/arlS/arlS/patA/patB	I	efrA/arlR/arlS/arlS/patA/patB
Enrofloxacin	S	efrA/arlR/arlS/gyrA/efrB/patB/soxS/efrA	S	efrA/arlR/arlS/arlS/patA/patB	I	efrA/arlR/arlS/arlS/patA/patB
Ciprofloxacin	S	efrA/arlR/arlS/gyrA/efrB/patB/soxS/efrA	S	efrA/arlR/arlS/arlS/patA/patB	R	efrA/arlR/arlS/arlS/patA/patB
Sulfafurazole	S	/	S	sul4	S	sul4
Sulfadiazine Sodium	S	/	S	sul4	R	sul4
Rifampin	S	rpoB/efrA/efrB/efrA/soxS	S	rpoB2/efrA	S	rpoB2/efrA
Clindymycin	S	lmrB/cfrA/lmrP/salA	S	lmrD/cfrA/lmrC/erm(45)/lmrP/vgaE/optrA	R	lmrD/cfrA/lmrC/erm(45)/lmrP/vgaE/optrA

### Biofilm formation assay

Microtiter crystal violet tests were used to measure the amount of biofilm that these three *S. equi* isolates formed. Even though these three isolates formed biofilms differently from one another, Figure 4 shows a link between *S. equi* strains’ biofilm production and antimicrobial potency. The HT1112 produces a lot of biofilms and has antibacterial properties (Figure 4B).

### Comparative genomic analysis of HT1112, HTP133, and HTP232

**FIGURE 5:**
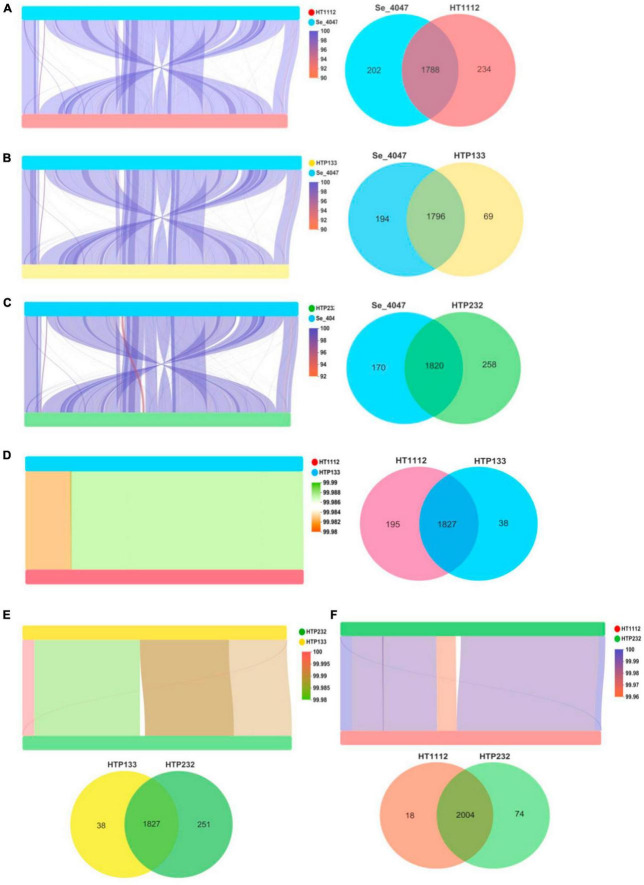
Linear comparison of *S. equi* isolate chromosomes and Venn diagrams illustrating overlap of proteins identified among **(A)** Se 4047 compared to HT1112, **(B)** Se 4047 compared to HTP133, **(C)** Se 4047 compared to HTP232, **(D)** HTP133 compared to HTP232, **(E)** HT1112 compared to HTP232, and **(F)** HT1112 compared to HTP133. Pairwise comparison of the chromosomes of **(A)** Se 4047 compared to HT1112, **(B)** Se 4047 compared to HTP133, **(C)** Se 4047 compared to HTP232, **(D)** HTP133 compared to HTP232, **(E)** HT1112 compared to HTP232, and **(F)** HT1112 compared to HTP133.

**FIGURE 6:**
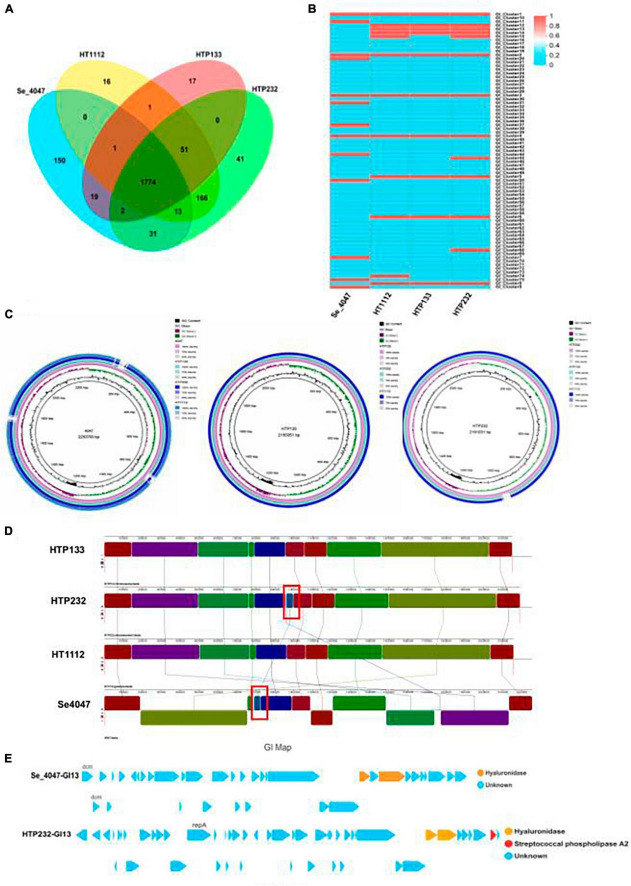
Comparison of three genomes of *S. equi* isolates to a reference strain. **(A)** The numbers of orthologous gene families and unique genes. The Venn diagram shows the number of orthologous gene families in the core genome (the center part) and the number of unique genes in each genome. The different colors indicated different sampling areas of the strains as indicated. **(B)** Heat map based on the comparison of genomic islands of three isolate genomes and a reference strain. **(C)** Alignment of *S. equi* HT1112, HTP133, and HTP232 genomes to those of reference strain Se 4047 using BRIG. **(D)** Pairwise comparison of the chromosomes of Se HTP133, Se HTP232, Se HT1112, and Se 4047 using Mauve. The image shown was generated by the Mauve rearrangement viewer. **(E)** The genomic island GI13 of Se 4047 and HTP232 harbored the hyaluronate lyase-coding gene *hya*.

We first observed a high degree of synteny was additionally for isolates HTP133, HT1112, and HTP232, suggesting a close relationship between these isolates (Figure 5). Genome synteny was further explored by comparing the linear organization of the chromosome of each *S. equi* isolate to *S. equi* 4047, and an increased number of genome inversions and rearrangements compared to *S. equi* 4047 were observed for isolates HT1112, HTP133, and HTP232 (Figures 5A, B, C). Since these isolates were from donkey cases, a high degree of global synteny and collinearity was observed among the HT1112 and HTP133 isolates (Figures 5D, E, F). Then we analyzed the shared and unique genes between isolates of HT1112, HTP133, HTP232, and *S. equi* 4047 (Figure 6). Core genomes are usually used to evaluate the genomic diversity within species. Our results showed 1,788 to 1,820 CDSs in the core genome were shared by these isolates; there were 69, 234, and 258 unique genes harbored by the HTP133, HT1112, and HTP232 strains, respectively. On the other hand, we investigated the shared and unique genes between isolates of HT1112, HTP133, and HTP232 (Figure 6A). There were 1,828 to 2,004 shared genes among them, and there were 18 to 251 unique genes harbored by these isolates, respectively. Compared to *S. equi* 4047, donkey isolates, HTP133, HT1112, and HTP232, were found to contain a higher number of genome rearrangements and inversions.

Genomic islands (GIs) are fragments of DNA derived from horizontal gene transfer between different bacterial genomes (Rodriguez-Valera et al., 2016) and play an important role in the virulence of *S. equi*. In this study, we identified 13 GIs in the HTP232 genome (Figure 6B). Using the IslandPath-DIMOB program (Bertelli et al., 2017), and for the entire chromosome of strains HTP133 and HT1112, the GIs were 10 and 12, respectively.

Visualization of the alignment of the *S. equi* and the predicted CDS regions of the three strains, HT1112, HTP133, and HT P232, using the BRIG is given in Figure 6C. An increased number of genome rearrangements and inversions were observed for isolates and *S. equi* 4047.

Genome synteny was further explored by comparing the linear organization of the chromosomes of three *S. equi* isolates to *S. equi* 4047 using Mauve [14]. Genome structure, which is shown in Figure 6D, A high degree of global synteny was observed among the donkey isolates HT1112, HTP133, and HTP232, with the exception of one insertion in the HTP232 genome.

The alignment of each *S. equi* predicted CDS to their closest relatives, *S. equi* 4047, is shown in Figure 6D. Multiple genome alignments of the *S. equi* strains HT1112, HTP133, and HTP232 and *S. equi* 4047 identified one distinct block of the genome that only exists in HTP232 and *S. equi* 4047 strains, comprising the hyaluronidase gene cassette.

### *Hya* on genomic island of *S. equi* isolates

The comparative genomic analysis revealed that the chromosomes of Se HTP133, Se HTP232, Se HT1112, and Se 4047 are generally collinear except for a genetic element transfer that we found between Se HTP232 and Se 4047 (Figure 6D). In Se 4047 GI13, the hyaluronate lyase-coding gene *hya* has been translocated to Se HTP232. Hyaluronate lyases are secreted enzymes that degrade HA and chondroitin, facilitating invasion by bacteria and their toxins. The transferred GI and hyaluronidase genes of Se 4047 were not conserved and consistent with those of the hyaluronidase genes in HTP232 (hyaluronidase gene, 1,112 and 1,883 bp) (Figure 6E).

Two CDS that code for hyaluronate lyase are present in the Se 4047-transferred GI 13 (26,322 bp). In addition, the two copies of hyaluronate lyase (761 and 1,883 bp) vary slightly in Se 4047. However, two distinct hyaluronidase genes (1,112 and 1,883 bp) are present in the transferred GI 13 (31,108 kb) of Se HTP232. Two distinct hyaluronate lyases have been acquired by Se HTP232 and are encoded on its GI 13 (Figure 6E).

**FIGURE 7:**
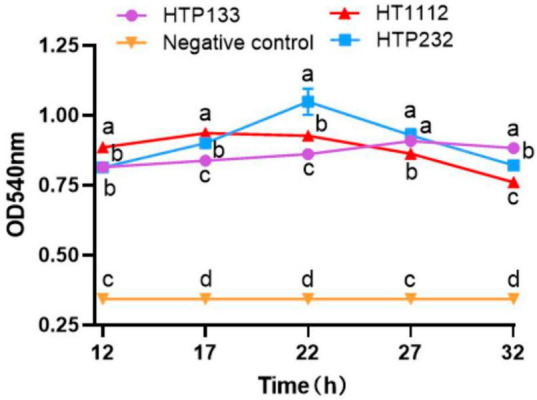
Hyaluronidase activity assay of 3 *S. equi* isolates. The enzyme activities were measured by monitoring the absorbance at 540 nm. Values represent the mean ± SD of three independent experiments. There is a significant difference (*p* < 0.05) between numbers with different letters; no significant difference (*p* > 0.05) exists between numbers with the same letter.

To demonstrate that the hyaluronate lyase phenotype was due to a gene, we grew Se HTP133, Se HTP232, and the Se HT11127 isolate to test the hyaluronidase activity. Thus, the phenotypic profiling results indicated that the Se HTP232 isolate plays an important role in the hyaluronidase activity of Se HTP232 (Figure 7).

## Discussion

Outbreaks of strangles in donkeys with severe clinical signs are becoming prevalent due to the increasing number of intensive donkey breeding farms in China (Dong et al., 2019). To date, there have been no published reports of genomic studies conducted on *S. equi* strains recovered from donkey farms in China. In this study, we determined the complete genome sequence of three donkey *S. equi* strains, HT1112, HTP133, and HTP232, isolated in China and used comparative genomic analysis to provide genetic evidence for their evolution. The whole genome sequence information of donkey-associated *S. equi* strains will contribute to the understanding of genetic features such as antimicrobial resistance and virulence-related genes.

*S. zooepidemicus* has been hypothesized to have developed from an ancestor strain of *S. equi*, which possesses just two sequence types (ST), ST179 and ST151, by MLST (Webb et al., 2008). Our findings indicated that *S. equi* 4047, these three donkey stains, and both sequence types ST179.

In this study, the complete genome sequence of *S. equi* strains HT1112, HTP133, and HTP232 was determined and compared to the reference genomes of *S. equi* strain 4047. Three donkey strain genomes were found to be highly colinear with each other, but compared to the reference genomes, there is a predominant rearrangement involving a lot of inversions by pairwise and reciprocal comparisons. In addition, the HTP232 strain has more unique genes compared with *S. equi* strain 4047. Holden et al. (2009) have reported that the pathogenic specialization of *S. equi* has been shaped by a combination of gene loss and gene gain through the acquisition of mobile genetic elements by comparing the complete genomes of *S. equi* strain 4047 and *S. zooepidemicus* strain H70.

The acquisition of prophage plays an important role in the evolution of many pathogenic bacteria (Brussow et al., 2004). The prophage was absent in the SzH70 genome, and unlike SzH70, Se 4047 is polylysogenic, containing four prophages (Beres et al., 2008). Here, our result revealed that the donkey strain HTP232 contains four prophages, and they could carry two genes (*hya*) through the prophages, which may increase its survival fitness (Beres and Musser, 2007). Going deeper into virulence characterization, HTP232 contains one more virulent factor than HT1112 and HTP133.

Concerning antibiotic resistance, these three donkey strains contain the same number of ARGs; however, based on the phenotype profile, the HT1112 exhibited enhanced antimicrobial resistance. It has been reported that bacteria within biofilms have an increased resistance to antimicrobials, and infections caused by biofilm-producing bacteria are frequently resistant to antibacterial chemotherapy (Jefferson and Cerca, 2006). Other researchers also revealed that *S. zooepidemicus* can form biofilms (Yi et al., 2014). In the present study, our results with the HT1112 isolate suggest that there is a correlation between biofilm formation and their capacity for antimicrobial resistance. Grenier et al. (2009) showed that a *Streptococcus suis* serotype 2 strain isolated from a case of meningitis in pigs could form a dense biofilm and suggested a correlation between biofilm formation and the establishment of infection. Moreover, bacteria with the capacity to colonize the host by forming biofilms have more advantages in establishing persistent infections (Falkinham, 2007). Therefore, the HT1112 isolate should be a potential candidate for our future investigation against *S. equi* persistent infections.

Hyaluronic acid (HA) is a linear anionic, non-sulfated, heteropolysaccharide composed of glucuronic acid and N-acetylglucosamine joined alternately by β glycosidic bonds (Marinho et al., 2021). Most bacterial hyaluronidase is believed to facilitate the pathogen’s invasion and survival in the host, which is critical to its pathogenicity and gives the pathogens an evolutionary advantage (Hynes et al., 2000; Desiere et al., 2001; Smith et al., 2005; Messina et al., 2016; Hu et al., 2021). It was reported that a few microorganisms of groups A and C *streptococci* are HA producers (Wei et al., 2012). The *S. zooepidemicus* H70 genome contains one single CDS encoding a hyaluronidase, whereas the *S. equi* 4047 genome has two hyaluronidase genes, and one hyaluronidase was encoded on a prophage (Waller and Robinson, 2013). This type of hyaluronidase was found to have a reduced substrate range and lower activity, which helps explain why *S. equi* infection rarely progresses beyond the lymphatic system (Lindsay et al., 2009). In the present study, consistent with the previous study findings in the horse isolate *S. equi* 4047, both the donkey strains HT1112 and HTP133 have one hyaluronidase encoded on a prophage. Interestingly, we note that HTP232 has two hyaluronidase genes carried on two different promoters, and one hyaluronidase gene contains a 6 bp deletion. Previous studies revealed that reduced hyaluronidase leads to high levels of hyaluronate capsules and enhances resistance to phagocytosis (Wibawan et al., 1999; Velineni and Timoney, 2015). The enhanced hyaluronate lyase activity may affect the levels of hyaluronate capsules in *S. equi* HTP232 and lead to low levels of capsules. Therefore, these results implied that the hyaluronidase gene gained by HTP232 might be critical to its pathogenicity, giving the pathogens an evolutionary advantage.

The genomic island GI13 of Se 4047 and HTP232 harbored the hyaluronate lyase-coding gene *hya*. The comparative genomic analysis of three donkey strains with Se 4047 revealed that there is horizontal gene transfer between the Se 4047 and HTP232 strains. It is worth noting that the exchange of genetic elements between host-restricted pathogens is rarely considered.

Reduced hyaluronate lyase activity provides an alternative explanation as to why *S. equi* maintains high levels of hyaluronate capsule, and in agreement with this, the Se HTP232 isolate of *S. equi* that was tested reduced virulence in a mouse challenge.

To sum up, this is the first publication that we are aware of that details the WGS and comparative analysis of the *S. equi* strain that was isolated from Chinese donkeys with strangles. The findings of our research have provided extensive insight into the phenotypic and genetic characteristics that may be used to distinguish between three isolates from donkey farms. Understanding how to prevent and cure infections may be made possible by genetic traits.

## Data availability statement

Original datasets are available in a publicly accessible repository: The original contributions presented in the study are publicly available. This data can be found in the NCBI repository under accession number: PRJNA1012111.

## Ethics statement

The animal study was approved by the Animal Ethics Committee, Xinjiang Agricultural University, China. The study was conducted in accordance with the local legislation and institutional requirements.

## Author contributions

YZ: Data curation, Investigation, Methodology, Writing – original draft. FL: Investigation, Writing – original draft. YS: Conceptualization, Funding acquisition, Project administration, Supervision, Writing – original draft, Writing – review & editing. HZ: Writing – original draft. BZ: Investigation, Writing – review & editing.
